# Papoid in Consumption

**Published:** 1893-11

**Authors:** E. A. Wood

**Affiliations:** Ex-Chairman of the Committee on Dietetics of the American Medical Association


### BUSINESS DEPARTMENT.

Address all letters relative to Business Matters and make all Money Orders payable to D. H. Howell, Business Manager.

Kindly remember that the Publishers must be notified by letter when a subscriber wishes his JOURNAL stopped. All arreages must be paid. The courts have decided that all subscribers to magazines and newspapers are held responsible until arrearages are paid, and their papers are ordered to be discontinued.

---

ATTENTION.—All communications and all matters pertaining to this department must reach us by the 20th of the month to insure insertion in the following month's issue.

---

### Special Notes.

SANDERS & SON'S EUCALYPTON EXTRACT (EUCALYPTOL.)—Whenever mention is made of "Oil of Eucalyptus" we beg you to bear in mind that such reference applies to our preparation, styled for distinction, "Eucalypti Extract (Eucalyptol)." To avoid disappointment, we would suggest to specify, when prescribing, our manufacture. Samples gratis through Dr. Sanders, Dillon, Iowa. Meyer Bros. Drug Co., St. Louis, Mo.,

### PAPOID IN CONSUMPTION.

BY E. A. WOOD, M. D.

Ex-Chairman of the Committee on Dietetics of the American Medical Association.

Knowing the power of papoid to destroy germs in ulcers and on open surfaces, I have employed it in ozena, ulcers of the larynx, and in cavities and ulcers in phthisis pulmonalis. I have used the drug, first by insufflation, but latterly by using the glycerole of papoid by the atomizer. Since the eight months of trial I have been more and more convinced of its efficiency in the lesions named.

The treatment followed is:

First.—Bromide of gold and arsenic internally, ten drops in water before meals.

Second.—Depress the tongue that the spray of papoid may thoroughly reach all parts of the larynx.

Third.—Cause the patient to breathe deeply that the drug may reach all parts of the bronchioles.

Fourth.—Employ the spray for at least ten minutes at each sitting.

Fifth.—Use the spray morning and evening.

If there is no ulcer the papoid can do no good used as a spray. To obtain the best results the glycerole of papoid should be diluted with an equal amount of alcohol.

When papoid is used as a digestive ferment in cases of consumption where there is debility, weak digestion and the suspicion of congested mucous patches, the drug should not be given in concentrated form, lest it dissolve the weakened tissues. In that case incorporate the papoid with the food before it is eaten. Sometimes it is better to partially prepeptonize the food.—*Pittsburg Medical Review, August*, 1893.

### SENNINE IN GYNAECOLOGY.

The greatest difficulty heretofore experienced in the treatment of cervicitis, abrasions, fissure and ulcerations of the os-uteri have been from the constant interference of the acrid discharges that effectually prevented any healing process. The various washes and solutions, owing to these poisonous discharges, have been only of temporary service. Sennine applied freely in its powder form coagulates the the oozing serum and thus hermetically seals the part until Nature restores its integrity. The application may be preceded by a thorough syringing with one part of Sennine to fifty of water. This solution is also very efficient in Leucorrhœa. If at the same time dessert spoonful doses of Dioviburnia be given internally three times daily the cure is very rapid and permanent.

DR. J. W. PRICE, of Atlanta, Ga., writes under date of Sept. 18th, 1893: "I have used Antikamnia in all caess of neuralgia and other painful neuroses, with decided success. I regard it as one of the best anodynes now in use by the profession."

